# Treating open lower limb fractures successfully; thoughts and current practice on therapy and centralization in The Netherlands

**DOI:** 10.1007/s00068-017-0874-7

**Published:** 2017-11-27

**Authors:** K. Oflazoglu, J. M. Hoogendoorn, P. van der Zwaal, E. T. Walbeehm, W. A. van Enst, H. R. Holtslag, D. Hofstee, P. Plantinga, M. Elzinga, H. Rakhorst

**Affiliations:** 1 Research fellow Orthopedic Hand Service, Orthopedic Surgery, Harvard Medical School, Massachusetts General Hospital, Yawkey Center, 55 Fruit Street, Boston, MA 02114 USA; 2 Department of Trauma Surgery, Haaglanden Medical Center, The Hague, The Netherlands; 3 Department of Orthopedic and Trauma Surgery, Haaglanden Medisch Centrum, The Hague, The Netherlands; 4 Department of Plastic and Reconstructive Surgery, Radboud University Medical Centre, Nijmegen, The Netherlands; 5 Kennisinstituut van de Federatie Medisch Specialisten, Utrecht, The Netherlands; 6 Department of Rehabilitation Medicine, Academic Medical Centre, Amsterdam, The Netherlands; 7 Department of Orthopedic Surgery, Noordwest Ziekenhuis Groep, Alkmaar, The Netherlands; 8 Emergency Department, Rijnstate Hospital, Arnhem, The Netherlands; 9 Department of Trauma Surgery, VU Medical Centre, Amsterdam, The Netherlands; 10 Department of Plastic and Reconstructive Surgery, Medisch Spectrum Twente, Enschede, The Netherlands

**Keywords:** Open lower limb fractures, Multidisciplinary treatment, National survey

## Abstract

**Introduction:**

The British Orthopedic Association (BOA) and British Association of Plastic, Reconstructive and Aesthetic Surgeons (BAPRAS) updated the evidence-based guidelines for the treatment and care of open lower limb fractures (BOAST 4). Following this, a Dutch version has been developed. The main points are multidisciplinary care, planning, and treatment of these injuries. Early osteosynthesis (within 7–14 days) combined with soft-tissue coverage results in more efficient care and less complications.

**Aim:**

To study the variation in treatment and thoughts among trauma, orthopedic, and plastic surgeons.

**Materials and methods:**

In this cross-sectional study 94 surgeons (57 trauma, 23 plastic, and 14 orthopedic surgeons) working at 46 centers completed an online questionnaire, consisting of 5 demographic, 14 hospital-related, 8 BOAST 4-related, and 2 centralization-related questions.

**Results:**

There was a strong agreement among surgeons about the best moment for multidisciplinary consultation, which was before initial debridement, while in practice, this often does not occur. All surgeons agreed that the initial debridement should be performed immediately by any surgeon, but not solely by trainees. Plastic surgeons responded that the definitive stabilization and wound cover should not exceed 7 days, while half of the trauma and orthopedic surgeons agreed that it should not exceed 14 days. Finally, most surgeons agreed that Gustilo 3 fractures should be centralized. However, there was disagreement on the need for centralization of Gustilo 2 fractures.

**Discussion:**

Surgeons agree on better and earlier multidisciplinary treatment of open lower limb fractures and the centralization of Gustilo 3 fractures.

## Introduction

Severe open lower limb fractures typically occur in young males as a result of a motorcycle accident [1]. These typical injuries are complex and patients often present with multiple injuries. As a result, prolonged treatment, hospital stay, and rehabilitation are characteristic in these people in the middle of careers and developing family lives. Although these injuries have a low incidence, all these factors result in high impacts on the patient life and psychology [2]. Treatment should, therefore, focus on decreasing treatment duration and postoperative complications to a minimum.

Medical infrastructure concerning these traumas has been set up in several countries. They aim to get severely injured patients appropriate care within the system, without transfer delay [3–7]. In 2009, the British Orthopedic Association (BOA) and British Association of Plastic, Reconstructive and Aesthetic Surgeons (BAPRAS) updated the evidence-based standards of care for the management of severe open lower leg fractures (BOAST 4) [8]. Early transfer and extensive teamwork and expertise between surgical and non-surgical specialties ensure a good clinical outcome. Several studies reported successful results of major trauma centres (MTC) in the United Kingdom following these standards. A decrease in treatment time, fewer required surgeries per patient, and a higher successful limb reconstruction rate have led to a decrease in deep infection rate [9–11].

A similar guideline is being developed in The Netherlands following the BOAST 4. With an estimated 580 new cases of open lower limb fractures in The Netherlands, almost half is classified as a Gustilo 3 fracture, based on the incidence of the UK [12, 13]. The Netherlands is a small country of 150 × 300 km and is densely populated with 17 million people and 134 hospitals with 91 emergency units. Infrastructure is so that in 2014 99.8% of the population lived within a 45-min ambulance ride from an emergency unit [14]. The guideline calls for early multidisciplinary care, planning, and treatment for these injuries. This would result in more combined approaches of osteosynthesis and soft-tissue coverage, improving outcome and reducing complications [15].

The aim of the present study was to study the current practice and therapy of these injuries in The Netherlands, with respect to the timing of multidisciplinary consultation (MDC), timing of debridement, target period for soft-tissue coverage, and centralizing these injuries.

## Materials and methods

### Study design

In this cross-sectional study, departments of orthopedic surgery, trauma surgery, and plastic surgery of 70 different hospital units in The Netherlands were contacted by e-mail between November 2015 and June 2016 to invite the surgeons to participate in this study.

All participants were asked to complete an online questionnaire to collect the following data: five hospital related (e.g., type of hospital, other specialists working at the hospital, how many colleagues in a partnership), 14 current treatment related (e.g., how many treated open tibial fractures per year, who participates in a multidisciplinary team when is the treatment plan discussed, time of definitive soft-tissue coverage), 8 BOAST 4 guideline-based questions, and 2 questions about centralizing these injuries.

Data were collected using SurveyMonkey (https://www.surveymonkey.com), an online data collection program. Most questions were on Gustilo 2 or higher, since Gustilo 2 fractures are frequently classified as Gustilo 3 after initial debridement [13].

### Statistical analysis

Results will be stated as frequencies and percentages. The difference in opinion about centralization of Gustilo 2 and Gustilo 3 fractures is analysed using the Wilcoxon matched-pairs signed-ranks test.

## Results

### Surgeon characteristics

Ninety-four surgeons returned the questionnaire: 57 trauma surgeons, 23 plastic surgeons, and 14 orthopedic surgeons, working at 46 different hospitals, a response rate of 60% for trauma surgery units, 40% of plastic surgery units, and 11% of orthopedic surgery units. Most surgeons (39%) worked in a top clinical hospital and in most cases (44%) a level I emergency department (Table 1). Four orthopedic surgery departments replied that open tibial fractures were not treated at their departments, and were excluded.

**Table 1:** Characteristics of the surgeons who completed the questionnaire about the current practice and therapy in The Netherlands

Surgeon characteristics	*n* = 94
	*n* (%)
Specialists	
Trauma surgeon	57 (61)
Plastic surgeon	23 (24)
Orthopedic surgeon	14 (15)
Hospital	
Academic	24 (26)
Top clinical	37 (39)
Rural	33 (35)
Emergency department	
Level I	41 (44)
Level II	33 (35)
Level III	20 (21)

### Hospital characteristics

The most noticeable results are described; see Table 2 in “Appendix” for all results. As mentioned, four hospitals replied that it did not treat or accept any eligible injuries, and these hospitals were excluded from the results. Most participants (> 70%) worked in a hospital with all team members of a multidisciplinary team hold practice. These specialties were defined as ER doctors, rehabilitation specialists, plastic surgeons with microsurgery facilities, trauma surgeons, and trauma-focused orthopedic surgeons.

**Fig. 1:**
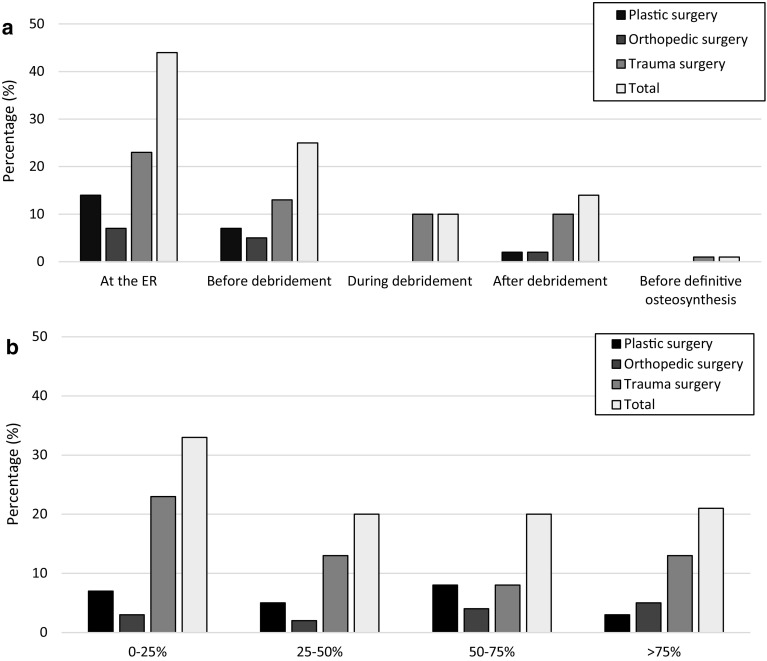
**a** Best moment for multidisciplinary consultation in case of a lower limb Gustilo 3 fracture. **b** In how many of the cases, multidisciplinary consultation takes place at the ER in case of a lower limb Gustilo 3 fracture

Seventy-three percent of the surgeons responded that they treat less than ten patients with a severe open lower limb fracture annually, whereas 7.6% treat more than 20 cases per year. According to 73% of the surgeons, the best moment for MDC in case of a Gustilo ≥ 2 fracture is prior to a first debridement. When asking for in which phase in their current practice this occurs, typically, MDC at the ER is not part of the standard procedures (Fig. 1).

Usually, the trauma or orthopedic surgeon has the lead after presentation of the patient at the Emergency Department (ED). This specialist can consult other specialties at his or her request. While most surgeons (71%) believe that MDC should occur before first debridement, almost half of the trauma surgeons replied that not everyone is available for MDC at night.

**Fig. 2:**
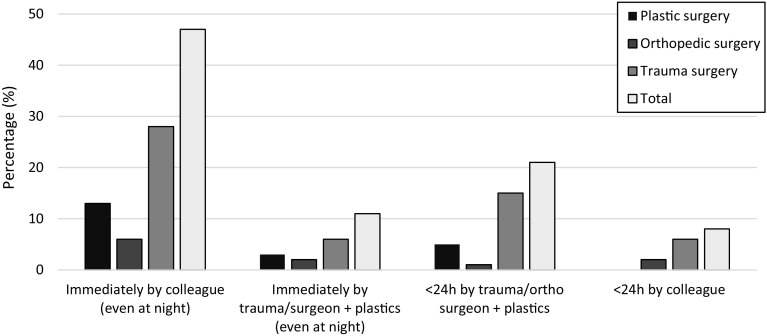
When and by whom first debridement should be done in case of a lower limb Gustilo 3 fracture

Two-thirds of the hospitals had a defined specialized multidisciplinary team. The UK guideline calls choosing for debridement by a specialized team over debridement as soon as possible. When asking surgeons on their opinion on timing of debridement, in the middle of the night or the next day by special-interest surgeons, 54% of all surgeons choose time over team, where debridement in the middle of the night by an on-call colleague is preferred. Only 24% believe that it should be performed within 24 h by a multidisciplinary team of plastic and orthopedic of trauma surgeon working together (Fig. 2).

The first debridement is usually performed by either the trauma or the orthopedic surgeon. In one-fifth of the cases, it is performed by the plastic surgeon.

According to 91% of respondents, negative pressure wound therapy is generally preferred not to be used longer than 2 weeks. Some surgeons specifically pointed out that this decision is multi-factorial and depends on issues such as size of the wound or logistical issues, e.g., availability theatre time for flap surgery.

**Fig. 3:**
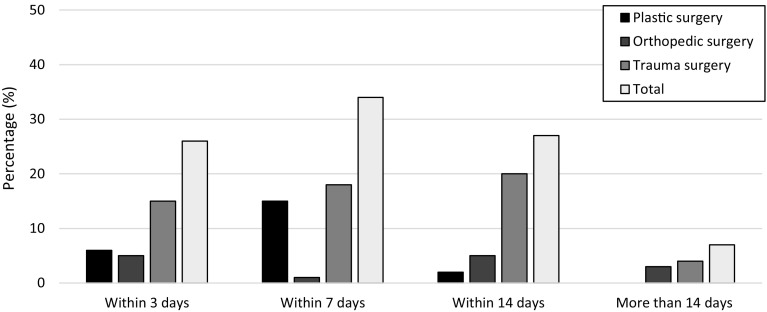
Target period for definitive skeletal stabilization when soft-tissue coverage is needed

Plastic surgeons keep 7 days as target period between time of injury and definitive osteosynthesis and soft-tissue cover. They prefer a combined approach instead of a staggered approach, where osteosynthesis and cover are separate procedures. More than 40% of the orthopedic and trauma surgeons have a longer target period of 14 days (Fig. 3).

**Fig. 4:**
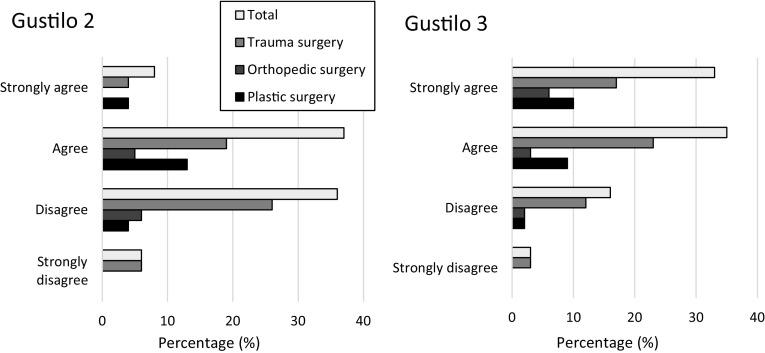
Opinions about centralization of open lower limb fractures

Finally, only 54%—mostly plastic surgeons—agreed that Gustilo 2 fractures should be centralized, while a significantly (*P* < 0.001) higher percentage of 79% of all surgeons agreed that Gustilo 3 fractures should be centralized (Fig. 4).

## Discussion

The goal of this paper was to have a proper assessment of the variation in the current perspectives and management of open lower limb fractures in The Netherlands using a national survey among plastic, orthopedic, and trauma surgeons. The majority of the surgeons agreed that the best moment for MDC in case of a Gustilo ≥ 2 fracture is prior to a first debridement. Half of the surgeons chose time over team, where debridement in the middle of the night by an on-call colleague is preferred. In one-fifth of the cases, it is performed by the plastic surgeon. More than 40% of the orthopedic and trauma surgeons have a target period of 14 days for soft-tissue coverage. Finally, four out of five surgeons agreed that Gustilo 3 fractures should be centralized.

In 2009, the BOAST 4 Standards were published which emphasized the importance of early transfer of patients with complex open lower limb fractures to a specialist centre [15]. In 2012, major trauma centres (MTC) and associated major trauma networks were created to improve patient transfer in the UK [9–11]. The major goal was reducing complications such as osteomyelitis by means of soft-tissue coverage within 72 h of injury.

There are three ED levels in The Netherlands, level 1 ED with the basic available care to level 3 ED with 24-h specialized care, 14 in total across the country. Several specialists are involved in treating open lower limb fractures: ED physicians, trauma surgeons, orthopedic surgeons, plastic surgeons, rehabilitation specialist, physiotherapists, and nurses specialized in wounds. ED physician informs the trauma or orthopedic surgeon about the arrival of complicated lower limb fracture. In general, either the trauma or orthopedic surgeon has the lead in the treatment and requests consultations of different specialists.

In the last decade, trauma systems have been established in several countries resulting in improved quality of care. In Ohio, USA, the mortality of trauma patients decreased by 40% after the establishment of a major trauma network [7]. Studies in Canada reported a decrease of 24–29% [4, 16]. Implementation of an inclusive trauma system in The Netherlands was associated with a substantial and significant risk reduction (16%) of death [3]. However, a Dutch national guideline has only recently (2017) been developed specific for the management of severe open lower limb fractures.

Ali et al. reported the outcomes of the treatment of open lower limb fractures after the centre was established as an MTC and compared these to the period before the establishment. The time from injury to soft-tissue coverage fell from 6 to 3.5 days, the time from definitive fixation to soft-tissue coverage fell from 5.0 to 2.0 days, and the deep infection rate fell from 27 to 8% [9]. Morrison et al. reported a decrease in required surgeries for patients that were directly admitted to the MTC, and underwent surgery only in the MTC in the Standards era (2011) compared to the Guidelines era (2006–2009) [10]. Wordsworth et al. reported a successful limb reconstruction rate of 98.5% and a deep infection rate of 1 in 65 in another MTC [11]. In our cohort, most surgeons were in favour of centralizing Gustilo 3 fractures.

A significant number of Dutch surgeons—mostly orthopedic and trauma surgeons—had a long target period for definitive skeletal stabilization if soft-tissue coverage is needed of more than 7 days. Exact rates are unknown, but it is estimated that significant number of these patients is not transferred quick enough to level I and II centres, with all essential specialists available 24/7, probably because of an initial underestimation of the injury severity. Most surgeons agreed that there should be a MDC in an early phase of treatment. Decreasing the treatment time in The Netherlands, by early transfer, a specialized multidisciplinary team, and a lower target period for soft-tissue coverage, might result in fewer required surgeries, higher limb reconstruction rate, and fewer complications.

The results should be interpreted in light of the strengths and limitations of the study. First, most responding surgeons worked in a hospital with a level I or II trauma level. Consequently, it is no surprise that these surgeons were agreeing on early transfer and centralization. Then again, many surgeons working in a rural hospital with a level III emergency department already do not treat these patients nowadays. Contrary to most other countries, both orthopedic and trauma surgeons treat bony injuries in The Netherlands. Second, some nuances are needed in generalizing these results. The answers on many of the questions about the current treatment depend on several factors, e.g., size of the wound, OR availability. Thus, surgeons opt for the best treatment for that patient, which means that some BOAST 4-related questions are hard to answer.

Even in a country like The Netherlands, where trauma care is well organized, the current treatment of complex open lower limb fractures in many hospitals is inadequate. Our results showed that The Netherlands should use their close network and infrastructure to aim to improve patient care and outcome for these patients. Moreover, the professionals from all disciplines seem to agree on steps to be taken such as centralization better interdisciplinary communication by defining teams and reduction of time between trauma and cover. An initial treatment by an adequate team within 24 h, instead of soon after arrival, should contribute to better outcomes. The results of this study will help the implementation of the Dutch guideline for the management of open lower limb fractures.

## Conclusion

This study clarified that the involved surgeons agree with the major components in managing the multidisciplinary therapy of open lower limb fractures. The importance of team work is essential and the Dutch surgeons agree on early transfer of the fractures to specialized trauma centres, and these are largely in accordance with the BOAST 4 standards.

This was the first step in implementing a new guideline. Our future aim is to compare the Dutch guideline with the original British guideline. Furthermore, we aim to assess the thoughts after implementing the Dutch guideline and compare those with the baseline results presented in this study. Eventually, we hope to get more insights on how to implement new medical guidelines in general for several European countries.

## Appendix

See Table 2.

**Table 2:** Results of the questionnaire about the current practice and therapy in The Netherlands

Results questionnaire	Plastic surgery	Orthopedic surgery	Trauma surgery	Total
Working specialist in center, *n* (%)				
General orthopedic surgeon	19 (83)	9 (69)	50 (89)	78 (85)
Orthopedic surgeon + trauma degree	20 (87)	13 (100)	32 (57)	65 (71)
General surgeon	19 (83)	9 (69)	40 (71)	68 (74)
Trauma surgeon	23 (100)	11 (84)	55 (98)	89 (97)
Plastic surgeon, no microsurgery	6 (26)	3 (23)	16 (29)	25 (27)
Plastic surgeon, with microsurgery	22 (96)	8 (62)	39 (70)	69 (75)
Rehabilitation specialist	22 (96)	8 (62)	50 (89)	80 (87)
Emergency department physician	21 (91)	10 (77)	48 (86)	79 (86)
Doctors in partnership, *n* (%)				
1	2 (8.7)	0	1 (1.8)	3 (3.3)
2	2 (8.7)	3 (23)	7 (13)	12 (13)
3	9 (39)	0	7 (13)	13 (17)
4	3 (13)	4 (31)	8 (15)	15 (16)
5	3 (13)	1 (7.7)	10 (18)	14 (15)
6	1 (4.4)	2 (15)	9 (16)	12 (13)
> 6	3 (13)	3 (23)	14 (25)	20 (22)
Lower leg surgeries per year, *n* (%)				
< 5	10 (43)	7 (54)	20 (36)	37 (40)
5–10	9 (39)	3 (23)	18 (32)	30 (33)
10–15	1 (4.4)	3 (23)	7 (13)	11 (12)
15–20	1 (4.4)	0	6 (11)	7 (7.7)
20–25	0	0	3 (5.4)	3 (3.3)
> 25	2 (8.7)	0	2 (3.6)	4 (4.4)
Specialists in MDC^a^, *n* (%)				
Trauma surgeon	22 (96)	10 (77)	56 (100)	88 (96)
Orthopedic surgeon	12 (52)	12 (92)	30 (54)	54 (59)
Plastic surgeon	18 (78)	8 (62)	36 (64)	62 (67)
Microbiologist	7 (30)	2 (15)	16 (29)	25 (27)
Rehabilitation specialist	14 (61)	3 (23)	31 (55)	48 (52)
Radiologist	0	0	0	0
MDC^a^ at the ED in case of Gustilo 2, *n* (%)				
0–25%	19 (83)	6 (46)	47 (84)	72 (78)
25–50%	2 (8.7)	3 (23)	2 (3.6)	7 (7.6)
50–75%	2 (8.7)	2 (15)	3 (5.4)	7 (7.6)
> 75%	0	2 (15)	4 (7.1)	6 (6.5)
MDC^a^ at the ED in case of Gustilo 3, *n* (%)				
0–25%	7 (30)	3 (23)	22 (39)	32 (34)
25–50%	5 (22)	2 (15)	13 (23)	20 (22)
50–75%	8 (35)	3 (23)	8 (14)	13 (21)
> 75%	3 (13)	5 (38)	13 (23)	21 (23)
Best moment for MDC^a^, *n* (%)				
At the ER	14 (61)	6 (46)	22 (39)	42 (46)
Before debridement	7 (30)	5 (38)	13 (23)	25 (27)
During debridement	0	0	10 (18)	10 (11)
After debridement	2 (8.7)	2 (15)	10 (18)	14 (15)
Before definitive osteosynthesis	0	0	1 (1.8)	1 (1.1)
Is everyone available for MDC^a^ (even at night)	19 (83)	9 (69)	32 (57)	60 (65)
Who decides amputation, *n* (%)				
Me	2 (8.7)	0	7 (13)	9 (10)
Together with trauma	21 (91)	7 (54)	45 (80)	73 (79)
Together with plastic	4 (17)	9 (69)	24 (43)	37 (40)
Together with orthopedics	4 (17)	5 (38)	5 (8.9)	14 (15)
Target period definitive osteosynthesis, *n* (%)				
< 3 days	12 (52)	3 (23)	17 (30)	32 (35)
< 7 days	11 (48)	3 (23)	19 (34)	33 (36)
< 14 days	0	7 (54)	20 (36)	27 (29)
Gustilo ≥ 2; Target period definitive osteosynthesis, *n* (%)				
< 3 days	10 (43)	2 (15)	8 (14)	20 (22)
< 7 days	12 (52)	2 (15)	11 (20)	25 (27)
< 14 days	1 (4.4)	6 (46)	30 (54)	37 (40)
> 14 days	0	3 (23)	7 (13)	10 (11)
Gustilo ≥ 2 + flap; Target period definitive osteosynthesis, *n* (%)				
< 3 days	6 (26)	5 (38)	15 (27)	26 (28)
< 7 days	15 (65)	1 (7.7)	17 (30)	33 (36)
< 14 days	2 (8.7)	4 (31)	20 (36)	26 (28)
> 14 days	0	3 (23)	4 (7.1)	7 (7.7)
Who will join definitive coverage, *n* (%)				
Trauma surgeon	21 (91)	5 (38)	54 (96)	80 (87)
Orthopedic surgeon	13 (57)	7 (54)	7 (13)	27 (29)
Plastic surgeon	23 (100)	13 (100)	55 (98)	91 (99)
Microbiologist	5 (22)	0	15 (27)	20 (22)
Rehabilitation specialist	6 (26)	2 (15)	17 (30)	25 (27)

^a^Multidisciplinary consultation
